# Chromosome Architecture and Genome Organization

**DOI:** 10.1371/journal.pone.0143739

**Published:** 2015-11-30

**Authors:** Giorgio Bernardi

**Affiliations:** 1 Science Department, Roma Tre University, Marconi, Rome, Italy; 2 Stazione Zoologica Anton Dohrn, Villa Comunale, Naples, Italy; University of Maryland School of Medicine, UNITED STATES

## Abstract

How the same DNA sequences can function in the three-dimensional architecture of interphase nucleus, fold in the very compact structure of metaphase chromosomes and go precisely back to the original interphase architecture in the following cell cycle remains an unresolved question to this day. The strategy used to address this issue was to analyze the correlations between chromosome architecture and the compositional patterns of DNA sequences spanning a size range from a few hundreds to a few thousands Kilobases. This is a critical range that encompasses isochores, interphase chromatin domains and boundaries, and chromosomal bands. The solution rests on the following key points: 1) the transition from the looped domains and sub-domains of interphase chromatin to the 30-nm fiber loops of early prophase chromosomes goes through the unfolding into an extended chromatin structure (probably a 10-nm “beads-on-a-string” structure); 2) the architectural proteins of interphase chromatin, such as CTCF and cohesin sub-units, are retained in mitosis and are part of the discontinuous protein scaffold of mitotic chromosomes; 3) the conservation of the link between architectural proteins and their binding sites on DNA through the cell cycle explains the “mitotic memory” of interphase architecture and the reversibility of the interphase to mitosis process. The results presented here also lead to a general conclusion which concerns the existence of correlations between the isochore organization of the genome and the architecture of chromosomes from interphase to metaphase.

## Introduction

The first breakthrough in our understanding of chromosome structure took place in 1968, when staining metaphase plant chromosomes with quinacrine mustard and ultraviolet light fluorescence microscopy showed bands that were characteristic of each chromosome pair [1], a result extended to human chromosomes shortly afterwards. In the following ten years, chromosomal banding at metaphase showed that the DNA of quinacrine (Q), or Giemsa (G), bands was GC-poor (as judged by its binding quinacrine), late-replicating, “condensed” and “inactive”, whereas the DNA of Reverse (R) bands was GC-rich, early replicating, “dispersed” and “genetically active” [2]. These remarkable early observations were later confirmed and extended, but metaphase chromosomal bands remained a “mystery” [3,4].

Chromosomal bands are, however, only one facet of a wider mystery which concerns chromosome architecture: how the same DNA sequences allow configurations as different as those of interphase and of mitosis; and how transitions can occur between the two configurations in both directions. The present work solved this mystery and revealed the organizational rules that are hidden behind the complexity of chromosome structure by applying a strategy, compositional genomics, to human chromosomes.

Compositional genomics, also born in 1968, was developed with the ambitious goal of obtaining an overall picture of genome organization. It is a minimalist, yet a very precise, quantitative strategy based on the most elementary and yet the most fundamental property of DNA, the frequency of short sequences and, as a proxy, base composition. Originally, this strategy was based on Cs_2_SO_4_ density gradient ultracentrifugation, as run in the presence of sequence-specific ligands, such as Ag^+^ [5]. Indeed, the classical CsCl ultracentrifugation, which fractionates DNA molecules on the basis of GC levels, does not have a satisfactory resolving power. In contrast, the Cs_2_SO_4_/Ag^+^ strategy could separate DNA fragments in a broad size range according to the different densities of the specific short sequences that bind the ligand. Needless to say, the compositional strategy was applied to genomic sequences as soon as they became available, in the late 1990’s. In fact, since short sequences determine the fine structure of the double helix as well as the interactions of DNA with proteins (*e*.*g*., histones to build nucleosomes, transcription factors to interact with regulatory sequences), compositional genomics is based on, and necessarily reflects, genome structure and function.

When applied to a mammalian genome, compositional genomics, in its original Cs_2_SO_4_/Ag^+^ ultracentrifugation version, not only separated satellite DNAs [5], as expected from their short-repeat structures, but also led to the discovery of an unsuspected compartmentalization of the genome into a small number of families of “main-band” (non-satellite) DNA molecules 10–20 Kb in size [6] that could not be resolved by CsCl ultracentrifugation. These families were characterized by different GC levels [6] and distinct short-sequence patterns [7], the latter being responsible for the separation. A compositional compartmentalization was then found in all eukaryotic genomes explored [8–13].

**Fig 1:**
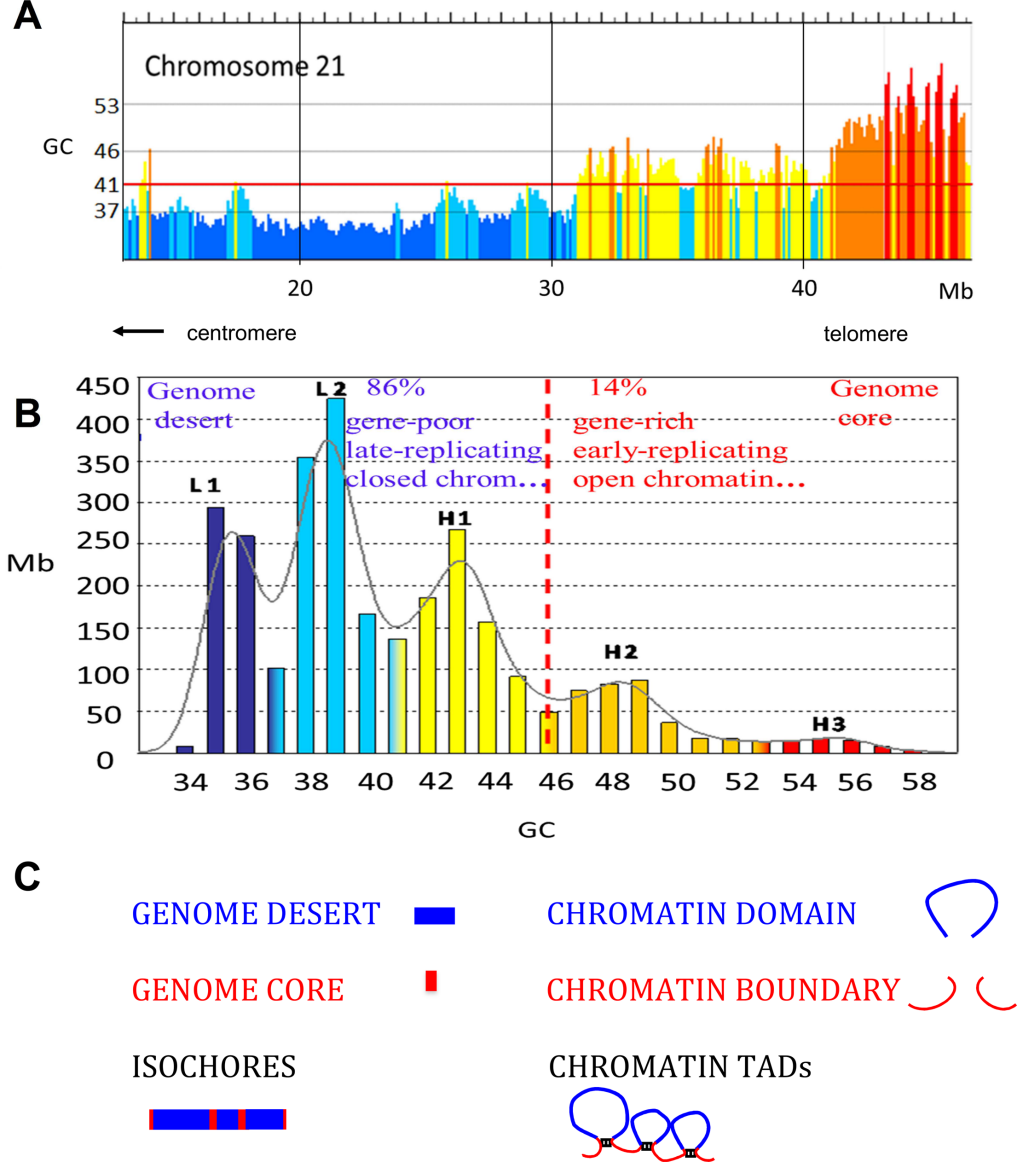
A. Compositional profile of human chromosome 21 (from the hg19 release) as seen through non-overlapping 100-Kb windows, using the IsoSegmenter program [15]. DNA stretches from isochore families L1 to H3 are represented here in different colors, deep blue, light blue, yellow, orange, red, respectively. The ordinate values are the minima GC values (valleys) between isochore families (see S1 Table). The red horizontal line at 41% GC separates the two (GC-poor and GC-rich) genome compartments. B. Isochore families. The histogram displays the isochores from the human genome as pooled in bins of 1% GC (modified from ref. [16]). The Gaussian profile shows the distribution of isochore families, which are represented in different colors as in Fig 1A. Gene densities (and all other structural and functional properties tested; see Table 1) define a genome desert, isochore families L1, L2, H1, and a genome core, isochore families H2, H3 (separated by a vertical broken red line). C. The scheme compares isochores belonging to the genome desert and to the genome core with chromatin domains and chromatin boundaries.

The 10–20 Kb DNA molecules mentioned above derived, in fact, by degradation during preparation from much larger DNA stretches, fairly homogeneous in base composition [14], that were called “isochores” for (compositionally) equal landscapes. Isochores can be visualized by looking at the GC profiles of chromosomal sequences by using a 100-Kb fixed window (see Fig 1A for chromosome 21, the smallest human chromosome, which comprises, however, isochores from all families).

Indeed, scanning base composition along the DNA sequences of human chromosomes revealed regions that 1) exhibit a fairly homogeneous base composition ([14,16]; see also S1 Table); 2) range from 200 Kb to several megabases [14,16]; 3) fall into five families, L1, L2, H1, H2 and H3 ([9]; also supported by the multimodal distribution of coding sequences [17]); 4) are flanked by sequences showing higher or lower GC levels, that generally belong to the compositionally closest families, so forming an ordered compositional mosaic; and 5) correspond to the families originally detected by ultracentrifugation [6,14,16]. The five isochore families are characterized by 1) increasing GC levels and GC ranges and decreasing average sizes ([16]; see Fig 1B and S1 Table); 2) different trinucleotide frequencies [18,19] and different nucleosome positioning patterns [20]; 3) increasing gene densities [21]. Segmenting human chromosomes on the basis of local GC levels provided a complete coverage of the human genome (hg17 release) with ~3,159 isochores having an average size of 0.9 Mb and totalling 2,854 Mb [16].

The interspersed isochores from the L1, L2 and H1 families and those from the H2 and H3 families define two “genome spaces” [22,23], a large “genome desert”, which is GC-poor, gene-poor, late-replicating, and characterized by closed chromatin, and a small “genome core”, which is GC-rich, gene-rich, early-replicating, and characterized by open chromatin (see Fig 1B, ref. [9] and S1 Table).

Isochores are only defined on the basis of the compositional properties of contiguous 100-Kb DNA stretches. However, all structural and functional properties of the genome that were tested are correlated with the GC levels of isochores [9,24,25], as shown by the non-exhaustive list of Table 1. This is an important conclusion in that it links genome properties with isochore composition. In other words, isochore maps of chromosomes are maps of structural and functional genome properties. Moreover, syntenic regions are characterized by similar isochore profiles ([26] K. Jabbari and G. Bernardi, paper in preparation).

**Table 1:** Structural and functional properties of the genome core *vs*. the genome desert ^(a)^.

**GC level +** [9]	**GC heterogeneity +** [16]^**(b)**^
**LINE density -** [28]	**Intron, UTR size -** [40]
**SINE density +** [29]	**Housekeeping genes +** [9]
**Gene density +** [21, 30]	**Developmental genes -** [41, 42]
**Chromatin** Open [31, 32]	**Reverse bands +** [16,43–45]
**Gene expression +**[33–36]	**Nuclear location** Central [31,32,46]
**4-strand +** [37,38]	**Translocations +** [47]
**B to Z +** [38]	**Breakpoints +** [48]
**Bendability +** [38]	**Fragile sites +** [49]
**Curvature -** [38]	**Proviral integration +** [9, 50]
**GC-rich trinucleotides +**[18,19]	**Insertions/deletions +** [51]
**CpG, mC +** [39]	**Recombination +** [52]
**CpG islands +** [39]	**Point mutations +** [53]
**Isochore size -** [16]	**Replication** Early [54–57]

(a) In general, the properties of the genome core are just opposite to those of the genome desert. +/- signs indicate positive/negative differences of the properties of the genome core compared to those of the genome desert. Nucleosome positioning patterns [20] and chromatin states [58,59] also differ in the two genome spaces (see Text).

(b) See S1 Table.

The correlations of GC levels 1) of coding sequences with contiguous non-coding sequences (introns and intergenic sequences, that represent 98.5% of the human genome); and 2) of isochores, isochore families and genome spaces with structural/functional properties, amount to a genomic code (a 25 year-old definition [27]; see also ref. [9]), the existence of which indicates that the genome is an integrated ensemble, a unit which obeys general rules. The final and crucial point of the genomic code, revealed by the present investigation, concerns the existence of correlations between the organization of the genome, as seen through its compositional properties, and the architecture of chromosomes from interphase to metaphase.

## Results and Discussion

### Interphase chromosomes and isochores

It is well established 1) that chromosomes occupy distinct territories in the interphase nucleus of eukaryotic cells [60]; 2) that GC-rich, early replicating, transcriptionally active chromatin regions are located in the nuclear interior [46]; and 3) that the gene-richest regions display a much more spread-out, open conformation compared to the closed one of the gene-poorest regions [31,32]. Moreover, super-resolution microscopy established the existence of a higher-order chromatin organization, the ~1Mb chromatin domains, that may comprise smaller sub-domains [60].

These results were confirmed and expanded through other approaches. Indeed, the development of chromosome conformation capture (3C) technology [61] and its variants led to a new insight into the three-dimensional chromatin organization of the interphase nucleus. The spatial proximity maps produced by Hi-C technology provided evidence for numerous domains that fall into two sub-chromosomal compartments, A and B, characterized, like the genome core and the genome desert, by open and closed chromatin, respectively [62–64].

Moving to a lower size range, it appears that the three-dimensional structure of chromatin at interphase begins to be well understood as the result of investigations on mammalian cells and *Drosophila* concerning: 1) the “lamina-associated domains” (LADs) and their borders [65,66]; 2) the different “chromatin states” of mammalian cells [58,59]; 3) the different “chromatin types” of *Drosophila* [67]; 4) the “topological domains” and the “topologically associating domains” (TADs) and their boundaries [68,69], as well as the corresponding “physical domains” of *Drosophila* and their borders [70,71]; and 5) the contact domains defined by the interaction patterns detected by *in situ* Hi-C ([72]; see refs. [73,74] for reviews).

While the focus of the investigations just mentioned concerned the connections between chromatin structure and gene expression/regulation, here the main interest was on the correlations between chromatin structure and compositional genome features in view of solving the mystery of the changes of chromosome architecture during the cell cycle. These correlations are briefly summarized in the following points.

1 The over 1,300 LADs (typically hundreds of Kb in size) of human interphase nuclei represent ~35% of the genome are characterized by a low density of transcriptionally inactive genes and by H3K27me3 histone modifications, and are demarcated by the sequence-specific, zinc-finger insulator protein CTCF (the CCCTC-binding factor). In fact, the borders of LADs are characterized not only by the presence of CTCF and H3K4me2, but also by a high gene density [66]. In human and mouse, constitutive LADs, cLADs, are characterized by long, GC-poor DNA stretches, whereas constitutive inter-LADs, ciLADs, show opposite properties, both features largely coinciding with isochore distribution [66].
2 In human T cells many different chromatin states were discovered and characterized [58]. These results, as analysed here, show that almost all (10/11) “promotor-associated” chromatin states correspond to H2 and H3 isochores (which only represent 14% of the genome), as also do many “transcription-associated” states (7/17) and “active intergenic” states (5/11). In fact, promotor- and transcription-associated as well as active intergenic states, except for very few borderline cases, are comprised within H1, H2 and H3 isochores. In contrast, all large-scale repressed and repeat-associated states correspond to L1, L2 and H1 isochores.
3 In *Drosophila*, five principal chromatin types were color-coded on the basis of their proteins [67]. Blue and green chromatins correspond to known heterochromatin types, marked by Polycomb/H3K27me3, and by HPI/H3K9me2, respectively. Black chromatin is the prevalent type of repressive chromatin at least in part under developmental control. Red and yellow chromatins are transcriptionally active euchromatins with high levels of histone modifications H3K4me2 and H3K79me3; they comprise different types of genes and replicate early in contrast with the other three types of chromatin.
4 In mouse embryonic stem cells about 91% of the genome are occupied by 2,200 topological domains of median size, 880 Kb, with a range of tens of Kilobases to several Megabases, separated by topological domain boundaries (76.3% of which are less than 50K in size) and “unorganized chromatin” (median size ~560 Kb) that are enriched in transcription start sites (TSS) as well as in housekeeping genes, tRNA genes and SINES [68]. In fact, the topological domains are not far in relative amount (91%) from the isochores of L1, L2 and H1 families that represent 86% of the human genome; also their median size, 880Kb, like that of the 1Mb domains [60], is comparable with the mean size of isochores, 0.9Mb, a value basically due to the predominance of isochores from L1, L2 and H1 families (see S1 Table). These are fair agreements, in view of the totally different approaches used and of the interspecies (mouse/human) genome comparisons. About 75% of the boundaries are demarcated by CTCF, but only 15% of all bound CTCF are located at boundaries, consistent with other roles played by CTCF, such as the stabilization of shorter-range intra-domain interactions [75]. These findings suggest that most topological boundaries only or mainly concern the very frequent intra-domain interactions; in addition, they are much too small in size to correspond to GC-rich isochores, whereas this correspondence is most likely in the case of the “unorganized chromatin”.

The “topological domains” [68] just described and the “topologically associating domains” (TADs) [69] are supported not only by similar results in *Drosophila* [70,71], but also by results indicating that TADs are stable regulatory units of replication timing and that the boundaries of replication domains can be identified with the boundaries of TADs [57]. This conclusion is in agreement with the previous identification of isochores with replication units [56] and with the connections between isochores and the topological domains mentioned above.

5 Recent work [72], using *in situ* Hi-C, in which DNA ligation is performed in intact nuclei, has shown that the human genome is partitioned into “contact domains” ranging in size from 40 Kb to 3 Mb (median size 185 Kb), which are associated with distinct patterns of histone marks (see below). About 10,000 loops were identified that frequently link promoters and enhancers, correlate with gene activation and show conservation across cell types and mammalian species. Loop anchors typically occur at domain boundaries and bind CTCF, predominantly in a convergent orientation, as well as cohesin sub-units RAD21 and SMC3. Interestingly, it was noted, “nearly all the boundaries observed are associated with either a sub-compartment transition (that occur approximately every 300 Kb), or a loop (that occur approximately every 200 Kb); and many are associated with both”.

Distinct pattern of histone modifications distinguish six sub-compartments: A1 and A2 (from the open chromatin A compartment) are both early replicating and gene-rich with highly expressed genes; A1, however, ends replication at the beginning of S phase, whereas A2 continues replicating into the middle of S phase; moreover, A2 has a lower GC level and contains longer genes compared to A1. The other three interaction patterns, B1, B2, B3, are correlated with loci in compartment B (characterized by closed chromatin) and show very different properties. Replication of B1 sub-compartment peaks during the middle of S phase, whereas sub-compartments B2 and B3 do not replicate until the end of S phase. Finally, sub-compartment B4 is only present in chromosome 19 and contains many KRAB-ZNF superfamily genes.

**Fig 2:**
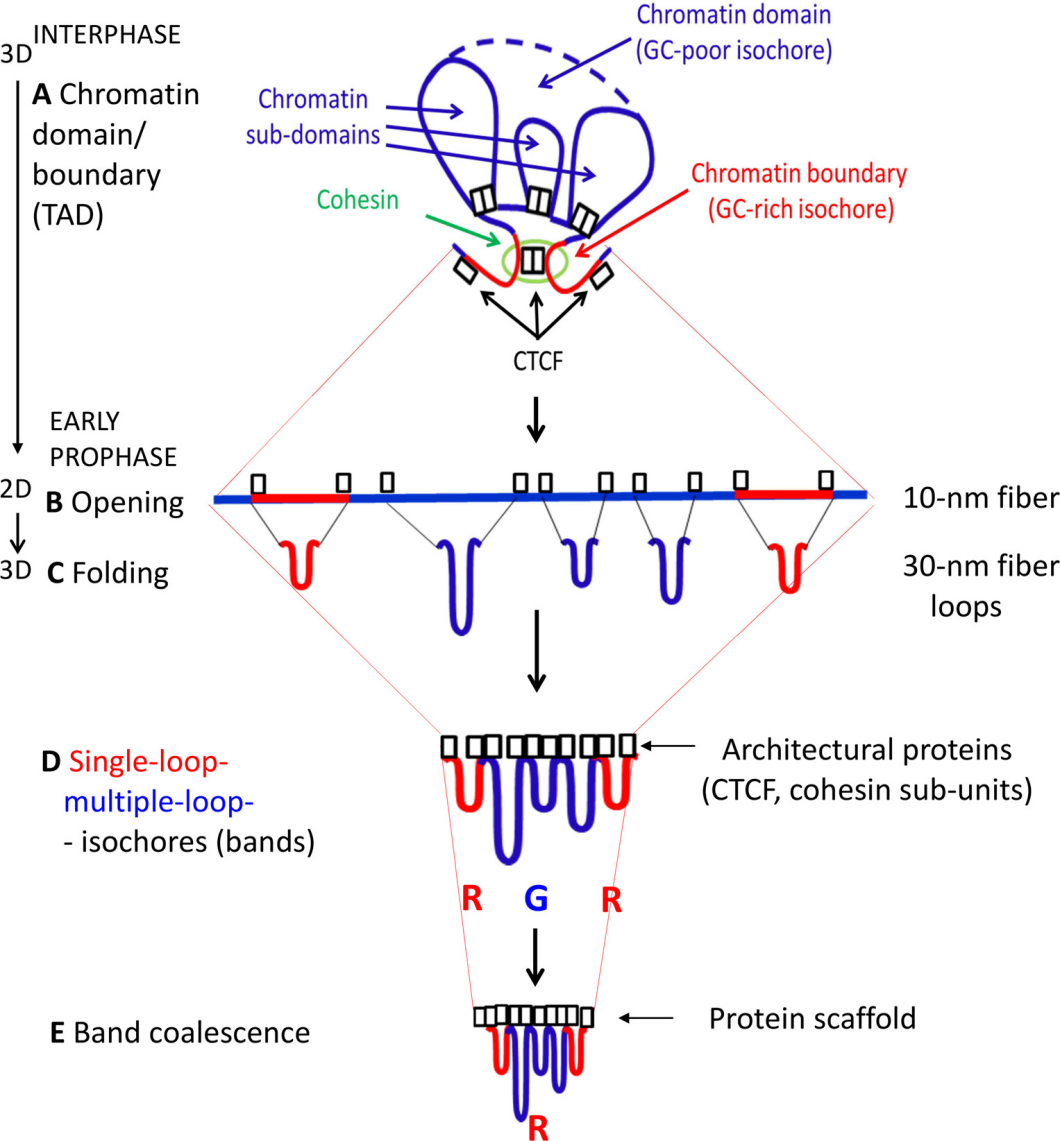
A. A scheme of an interphase chromatin loop (a topologically associating domain, TAD, with three sub-domains in this figure). The DNA framework of the loop is a large GC-poor isochore. The loop is closed by anchors (chromatin boundaries) that interact with two architectural proteins, CTCF (boxes) and cohesin (green oval). A number of sub-domains have their loops anchored by CTCF and cohesin sub-units (boxes) (see Text). B. Opening of the three-dimensional architecture of the domains and sub-domains in a linear chromatin structure, possibly in a “beads-on-a string”, 10-nm conformation. Architectural proteins are visualized as still linked to their binding sites (see Text). C, D. Folding of the open structure into 30-nm fiber loops anchored by the architectural proteins and compaction into three early prophase, single-isochore bands R-G-R, the central one being a multiple-loop band, the flanking ones single-loop bands. E. Coalescence of single-isochore bands into multiple-isochore bands. In the example shown, the R-G-R single-isochore bands coalesce into an R band because of a “majority rule” (2 R *vs*. 1 G). Architectural proteins form a discontinuous protein scaffold of the chromosome (see Text).

The architecture of interphase chromatin may be schematically visualized, at least for the purpose of the present work, as a set of looped domains and boundaries (see Fig 1C). While domain boundaries generally correspond to (short) GC-rich isochores anchored by architectural proteins, such as CTCF and cohesin (present as a ring structure or as cohesin sub-units), looped domains correspond to (long) GC-poor isochores. In turn, looped domains essentially consist of sub-domains, most of which are anchored by CTCF and by cohesin sub-units (as shown in Fig 2A).

The results presented so far are compared in Table 2 with the properties associated with the genome core and the genome desert. (In fact, several properties of the genome core listed in Table 1 could be added to the right column of Table 2). This comparison leads to the conclusion that the properties of compartments and sub-compartments, as well as those of chromatin domains and boundaries, match those of the isochores from the genome desert and the genome core, respectively, in spite of not always completely overlapping with each other because defined on the basis of different approaches.

**Table 2:** Isochores & interphase chromatin^(a)^.

**ISOCHORES [9]**	
**Genome desert (L1, L2, H1 families).**	**Genome core (H2, H3 families).**
**Gene-poor. LINES.**	**Gene-rich. SINES.**
**Closed chromatin.**	**Open chromatin.**
**Late replicating.**	**Early replicating.**
**86% of the genome.**	**CpG islands. HK genes** ^(a)^ ^.^
**(av. size 0.9Mb)**	
**COMPARTMENTS [62]**	
**B: closed chromatin**	**A: open chromatin**
**DOMAINS AND BORDERS/BOUNDARIES**	
**Domains:**	**Borders:**
**LADs, lamina- associated domains [65]**	**CTCF, promoters, CpG islands**
**gene-poor, H3K27me3**	**gene-rich, H3K4me2**
**cLADs, constitutive LADs, GC-poor [66]**	**ciLADs, constitutive inter-LADs, GC-rich**
**Topological domains [68]**	**Boundaries +**
**91% of the genome**	**“unorganized chromatin”**
**(av. size 0.88Mb)**	**(av. size 0.56 Mb)**
**Topologically associating domains TADs[70]**	**tRNAs, SINES, TSS** ^(a)^
	**Housekeeping genes**
**Physical domains** ^**(b)**^ **[71,72]**	**Boundaries**
**repressive marks**	**active marks**
**CHROMATIN STATES [58]**	
**Large-scale repressed**	**Promoter-associated**
**Repeat-associated**	**Transcription-associated**
	**Active intergenic**
**CHROMATIN TYPES** ^**(b)**^ **[67]**	
**Blue/Green:**	**Red, Yellow:**
**Heterochromatin**	**active chromatin**
**Polycomb/H3K27me3, HPI/H3K9me2**	**H3K4me2, H3K79me3**
**Black: repressive chromatin**	**both early replicating**
**all late replicating**	
**SUB-COMPARTMENTS** ^**(c)**^ **[72]**	
**B1, H3K27me3; facultative heterochromatin; replication to middle S phase, B2,B3, replication to end S phase**	**A1,A2, gene-rich, high expression, H3K36me3, H3K79me2, H3K27ac, H3K4me1, both early-replicating** ^**(c)**^

^(a)^HK, housekeeping; TSS, transcription start sites.

^(b)^ *Drosophila*. Other data concern mammalian cells.

^(c)^see also Text.

Finally, the chromosome architecture is conserved across mammalian species [72]; and in syntenic regions [76]. These observations parallel the conservation in mammalian genomes of isochore families [10], and of compositional landscapes of syntenic regions [26]. Altogether, these findings indicate a correlation between interphase chromatin architecture and the corresponding compositional landscapes (K. Jabbari and G. Bernardi, paper in preparation).

### Interphase chromatin to prophase bands

Many years ago, evidence was presented showing that GC-poor and GC-rich isochores are associated with the G and R metaphase bands of vertebrate chromosomes, respectively [22]. This association could not, however, be a simple one since isochores are much smaller than the DNA sequences of metaphase bands.

An approach was developed in order to understand this complex connection, compositional mapping [9, 24, 25]. This approach was initially based on assessments of GC levels around genome landmarks (*e*.*g*., genes localized on the physical map) of metaphase chromosomes [9], then on *in situ* hybridization of DNA from L1 and H3 isochores on metaphase and prometaphase chromosomes [9, 43, 44], and, finally, on human genome sequences [9, 16, 45]. The latter results provided detailed information on the sizes and GC levels of isochores, of prometaphase bands and of metaphase bands for all human chromosomes. Here, a new analysis of the Supplementary data (~120 pages) of these investigations [16, 45] was done, leading to new results and to a model for the critical transition from interphase chromatin to early prophase bands, which is presented below.

**Fig 3:**
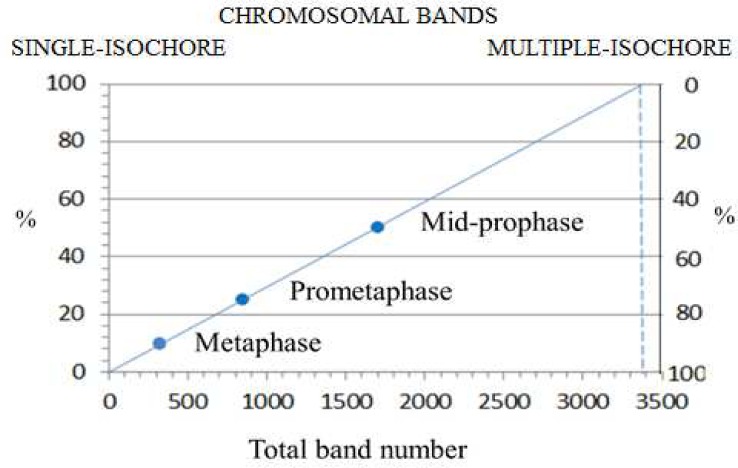
The percentages of single-isochore bands are plotted against the total number of bands at metaphase (400 bands), prometaphase (850 bands) and mid-prophase (1,700 bands) and extrapolated to 100% single-isochore bands.

1. At the beginning of mitosis the three-dimensional organization of interphase chromatin disappears [64], as expected, and is replaced in early prophase of human chromosomes by over 3,000 bands [77]. This number approximately matches the number of isochores, ~3,200. This preliminary indication that early prophase bands may correspond to individual isochores [16] is now definitely supported by the observation (Fig 3) that single-isochore bands represent ~8% of metaphase bands, ~25% of prometaphase bands and ~50% of mid-prophase bands (in chromosome 1, for example, the 122 bands of mid-prophase [77] correspond to 233 isochores; ratio = 0.52). Indeed, the three relative amounts just quoted indicate, by extrapolation, that single-isochore bands represent the totality of early prophase bands when the number of the latter is ~3,400, a value close to the total number of isochores (see Fig 3).
2. The very early decrease in band numbers in early prophase (see Fig 3) indicates a coalescence of the 30-nm fiber loops (a conventional definition because of a current debate) that form the bands. Obviously, the transition from the loops of interphase chromatin to the 30-nm fiber loops of early prophase needs a transient intermediary step. This can be visualized as the opening up of the three-dimensional structure of interphase chromatin (Fig 2A) into an extended, essentially two-dimensional chromatin configuration, probably a 10-nm “beads-on-a-string” structure (the “beads” corresponding to nucleosomes), in which GC-rich and GC-poor isochores alternate (Fig 2B). In the example of Fig 2, the anchors of an interphase chromatin domain (Fig 2A) are opened up into two (short) GC-rich isochores flanking a (long) GC-poor isochore, the cohesin ring, if present, also being opened into its sub-units. This opening process also concerns the anchors of the sub-domains and the corresponding architectural proteins, CTCF and cohesin sub-units (Fig 2B).
3. The folding process of the extended chromatin structures into the 30-nm fiber loops just described does not take place at random locations on chromosomes. Indeed, the alternance of GC-rich and GC-poor bands in early prophase indicates that the folding involves GC-rich and GC-poor isochores, respectively. This raises a question, concerning which signals demarcate the GC-rich and the GC-poor of the extended chromatin structures. The answer proposed here is that the signals are the same architectural proteins that were demarcating the anchors of the loops, CTCF and cohesin sub-units, and that are still associated with their binding sites in mitotic chromosomes. This demarcation seems to apply differentially to the architectural proteins located at domain boundaries and to those that are associated with sub-domains. In the first case (see Fig 2), the two GC-rich isochores of the domain boundary may be folded in single loops, whereas the GC-poor isochore of the chromatin domain is folded in multiple loops that originate from the sub-domains (see Fig 2C and 2D).
4. The single-isochore bands (whether single- or multiple-loop) start coalescing very early into multiple-isochore bands, as indicated by the early decrease of band numbers (see Fig 3). This coalescence appears to follow a precise rule in that it involves an odd number of single-isochore bands (most often three, Fig 2E), as shown by the persisting GC alternation of bands in the increasing number of multiple-isochore bands. The bands originating by the coalescence process just mentioned will be G or R bands according to a “majority rule”, namely according to the number of G or R bands in the coalesced bands (see Fig 2E).
5. In this model, the architectural proteins, CTCF and cohesin sub-units, are retained after the interphase to mitosis transition, like a number of other proteins (see a later section). This model is interesting for three main reasons: (i) the 30-nm fiber loops of chromosomes were estimated to be in the 100 Kb range, a range that is approached by the median size of all chromatin loops, 185 Kb [72]; (ii) the band coalescence may be driven by the increasing interactions among the architectural proteins, that, in fact, contribute to form the protein scaffold of mitotic chromosomes; (iii) the model explains the recovery of the original three-dimensional architecture of interphase chromatin at the exit from mitosis.

### Prophase to prometaphase bands

**Fig 4:**
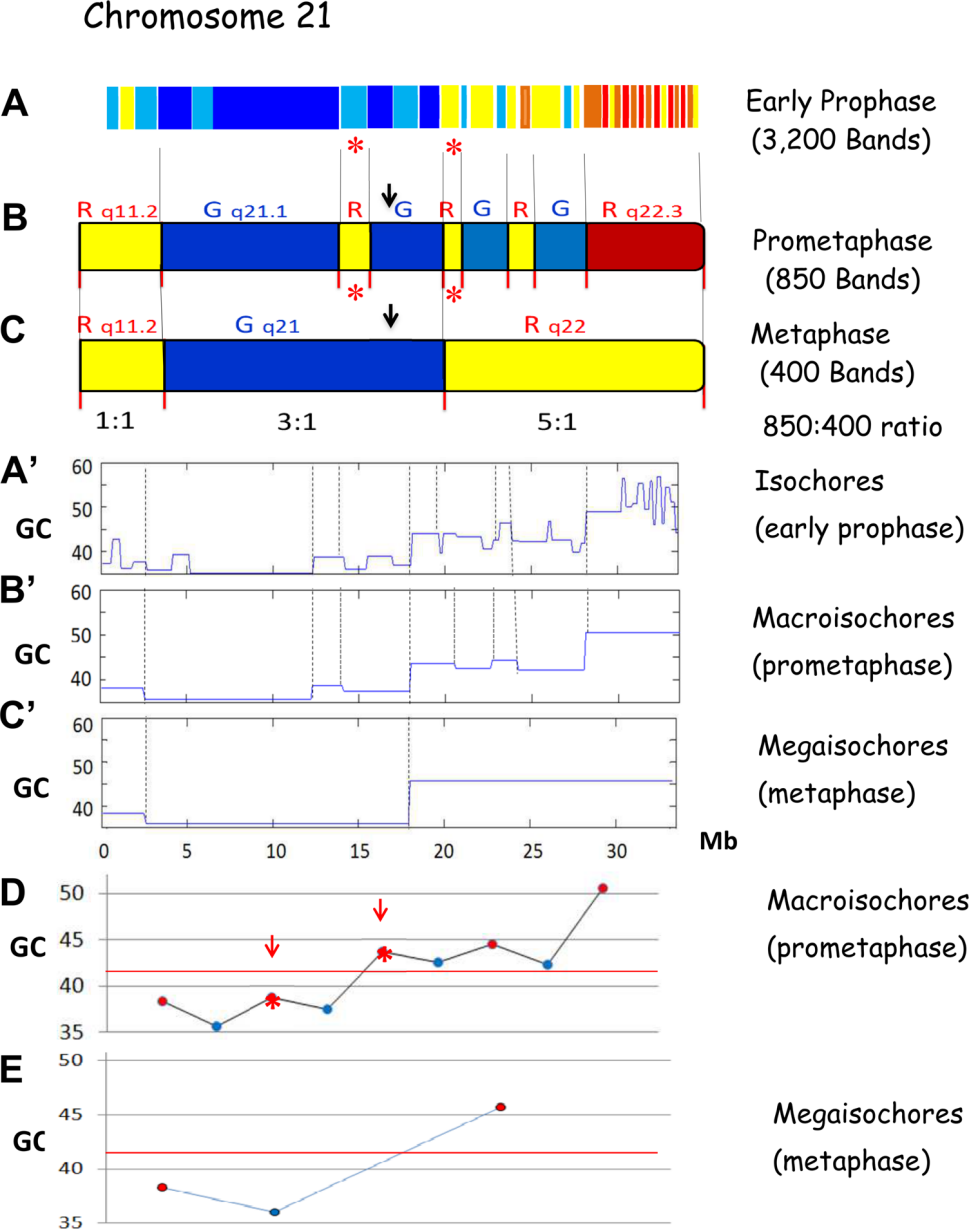
The banding pattern of chromosome 21: (A), at early prophase, (B), at prometaphase and (C) at metaphase. Vertical lines connect early prophase bands formed by single isochores (marked by red asterisks) or isochore blocks (the macroisochores) with prometaphase bands. B→C. The following coalescence process leads to different ratios of prometaphase to metaphase bands, 1:1, 3:1, 5:1. A’ B’ C’. The compositional profiles A^’^ of isochores (early prophase); B^’^ macroisochores (prometaphase) and C^’^ megaisochores (metaphase). D, E. GC levels of prometaphase (D) and metaphase (E) bands. Blue and red points indicate G and R bands. Red arrows and asterisks indicate single-isochore bands. The red horizontal line separates the two genome compartments, GC-poor and GC-rich.

Fig 4A and 4B shows the transition, in chromosome 21, from early prophase bands to prometaphase bands. In two cases (bands q21.2 and q22.11), prometaphase bands correspond to single isochores, in all other cases to multiple contiguous isochores, to isochore blocks (the macroisochores). At prometaphase, multiple-isochore bands represent 75% of all bands (see Fig 3).

**Fig 5:**
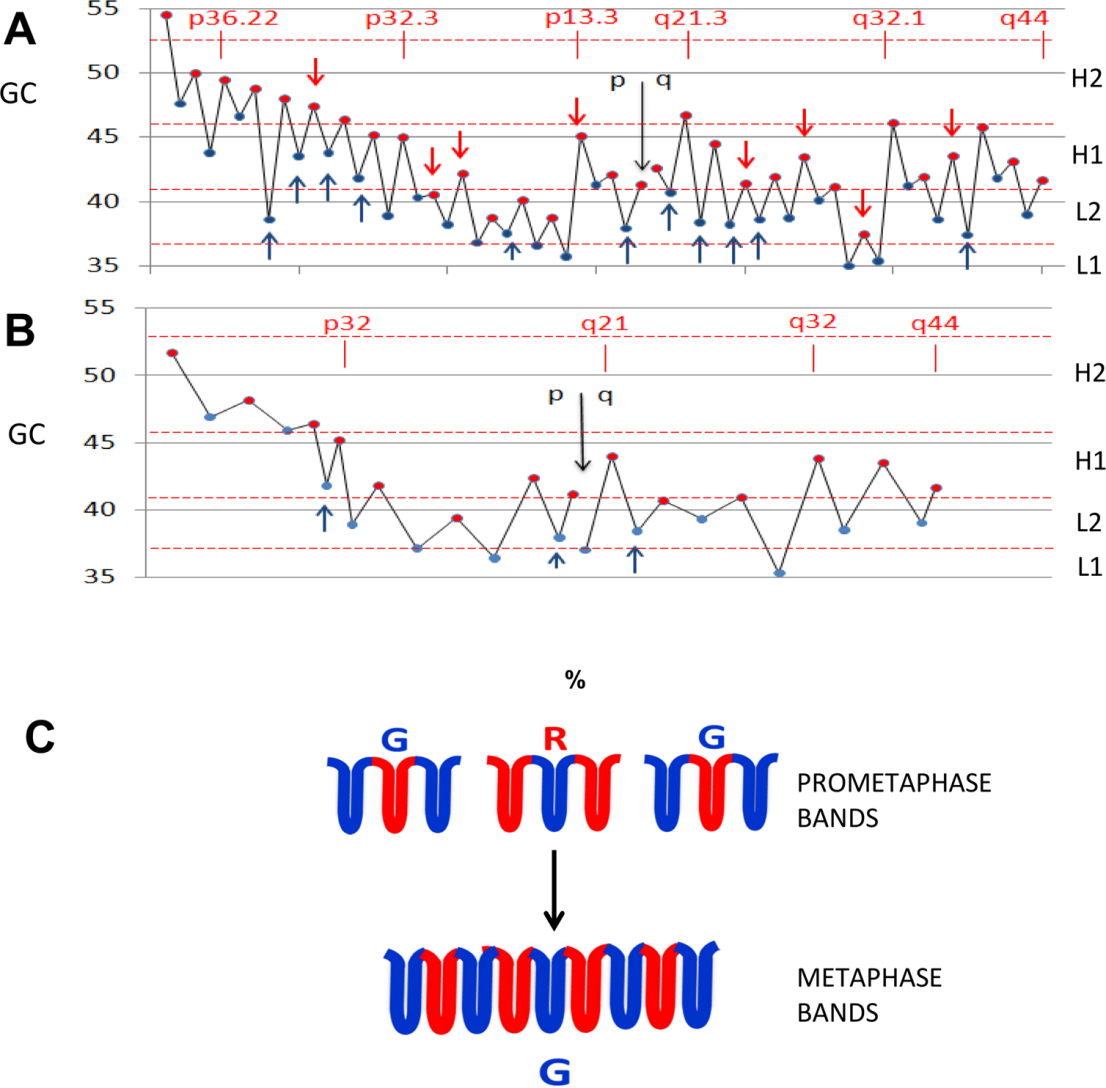
GC levels of prometaphase (A) and metaphase (B) bands of chromosome 1. Black arrows indicate p/q arms intervals, blue and red points indicates G and R bands, arrows single-isochore bands. Horizontal broken lines indicate the GC boundaries of isochore families. C. Scheme of the coalescence of prometaphase into metaphase bands.

An important feature of prometaphase bands is that not only their isochores and macroisochores alternate between higher and lower GC levels, but also that these levels are different in different sub-chromosomal regions, the compositional compartments. For example, the first two R bands on the centromeric side of chromosome 21 are lower in GC than the last two G bands on the telomeric side (see Fig 4D; see also Fig 5A for chromosome 1). These results lead to three conclusions: 1) GC contrasts and not absolute GC values are responsible for banding; 2) contrasts may be stronger or weaker (see Fig 4D), in agreement with the different degrees of staining intensity (G1 to G4) already noted for G bands [78]; 3) compositional compartments correspond to several prometaphase (and metaphase) bands. In fact, GC-rich and GC-poor compartments also alternate and tend to be located in telomeric and centromeric regions; interestingly, they show some profile similarity to the A and B compartments [64], respectively.

### Prometaphase to metaphase bands

Fig 4B and 4C display the bands of human chromosome 21 from prometaphase to metaphase. Three different situations were found, in which the ratios of prometaphase to metaphase bands are 1:1, 3:1 and 5:1. While the third situation is very rare [45], the second one is only slightly more frequent than the first one, so accounting for the overall 850/400 ratio of ~ 2:1. In other words, the transition from prometaphase to metaphase bands essentially is a coalescence process in which about half of prometaphase bands coalesce in a 3:1 ratio into metaphase bands, the other bands remaining as they were at prometaphase, except for a likely further compaction.

The 400-band ideogram shows, therefore, the existence of another level of chromosome organization (also following the “GC alternation rule”), in which next to the prometaphase bands that have not changed at metaphase, there are other ones that derive from a coalescence process. This involves tighter contacts within sets of contiguous prometaphase bands that correspond to macroisochore blocks, the megaisochores (another new term). Fig 4A’, 4B’ and 4C’ display the compositional profiles of isochores, macroisochores and megaisochores of chromosome 21. Fig 4E and Fig 5B show the GC levels of metaphase bands of chromosome 21 and 1, respectively. Fig 5C presents a scheme of the process leading from prometaphase to metaphase bands. Interestingly, at resolutions below the standard 400 bands (for instance at 200-band resolution), banding increasingly tends to correspond to compositional compartments.

### Models for metaphase chromosome structure

The most widely accepted model of metaphase chromosome structure is the “loops-on-a scaffold” model [79], originally derived from the electron microscopy observation of a residual metaphase-shaped structure in histone-depleted metaphase chromosomes. In this model [80], 1) metaphase chromosomes have a central protein network, a “scaffold”, which interacts with “scaffold associated regions”, the SARs (or MARs, matrix attachment regions), which are AT-rich (<35% GC) DNA regions stretching from 0.7 Kb to several Kilobases; and 2) bands arise from a differential folding path of the highly AT-rich regions, in which “R bands do not simply represent, as it is classically assumed, a homogeneous AT-depleted chromosomal disk, but, rather, contain a central AT queue linking adjacent G bands” [80].

**Fig 6:**
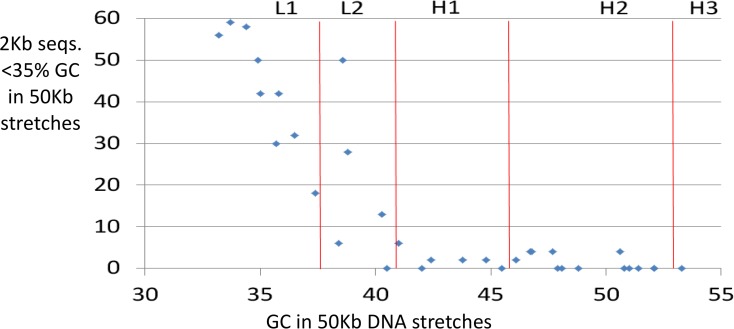
Amounts of 2-Kb sequences <35% GC as present in 50-Kb stretches of chromosome 21 are plotted against the GC levels of the 50-Kb stretches. Vertical red lines indicate the borders of isochore families.

Recent investigations have shown, however, the existence of two problems with this model: 1) the complete disintegration of metaphase chromosomes upon the action of micrococcal nuclease shows that metaphase chromosomes do not have a continuous protein scaffold [81]. 2) AT-rich (<35% GC) regions of 0.7 to several Kilobases practically do not exist in the isochores of the H1, H2, H3 families that form the R bands (see Fig 6). In chromosome 1, only one R band out of fourteen (0.7% of all R bands) is below 41%GC, the border between L2 and H1 isochores (see Fig 5B). The latter point raises an insoluble problem for the model [81], because the regular existence of AT-rich queues in R bands of chromosomes is not supported.

Interestingly, the model of metaphase chromosomes presented in the preceding section still is a “loops-on-a scaffold” model, in which, however, the chromatin loops are linked to the scaffold by the architectural proteins, themselves part of the scaffold. The progressive compaction from prophase to metaphase bands can be visualized as due to the coalescence of consecutive 30-nm loops (in agreement with ref. [64]), a process possibly driven by the increasing interactions of the discontinuous scaffold of architectural and other proteins. Condensins I and II also participate in this compaction which provides rigidity to chromosomes and helps the disentanglement of chromatids [82].

### Metaphase to interphase

While the transition from the three-dimensional architecture of interphase chromosomes to the increasingly compact one of metaphase chromosomes can be (wrongly) visualized just as a stochastic folding process, the reverse transition from metaphase to interphase is much more difficult to explain in simple terms. The reason is that the architecture of the new interphase chromosomes is such as to allow a quick reactivation of the original cell-specific programs [83–88], which implies that the original chromatin loops and anchors are precisely reformed. In fact, recent investigations have shown how sensitive chromatin functions are to changes in interphase architecture [87,89].

The model presented in Fig 2 can, however, solve this problem if read in the reverse from mitotic to interphase chromosomes. Needless to say, this explanation relies on the idea that architectural proteins, such as CTCF and cohesin sub-units remain associated with chromosomes during mitosis, in which case they fulfill a role in the folding of 30-nm fiber loops into isochores (see a preceding section). This idea is totally acceptable if one takes into consideration a number of recent results. Indeed, it has been shown that not only H3K9me3, H3K27 and polycomb group proteins, but also a fraction of transcription factors and of chromatin binding proteins are retained in mitotic chromosomes [83–88]. Moreover, there is evidence along this line for cohesin as well [90].

It is then reasonable to think that the “mitotic memory” of the interphase architecture concerns the entire three-dimensional architecture of interphase chromosomes which allows the same cell-specific expression programs of the mother cells to be achieved thanks to the conserved link between architectural proteins and the corresponding binding sites on DNA.

## Conclusions

These investigations lead to two major conclusions. The first one concerns the explanation of the reversible changes of chromosome architecture through the cell cycle. The second has to do with the connection between genome organization and chromosome architecture.

Our understanding of chromosome architecture at interphase has recently made remarkable advances, essentially thanks to the development of chromosome conformation capture (3C) and derived approaches. In contrast, the old mystery surrounding the transitions of chromosome architecture from interphase to mitosis and from mitosis to interphase at the beginning of the following cycle, has been waiting for a solution for a long time.

This mystery has now been solved using a strategy which took advantage of our previous knowledge of genome organization in a critical size range which encompasses isochores, interphase chromatin domains and boundaries and chromosomal bands. A key point was to understand that the same architectural proteins, such as CTCF and cohesin sub-units, could not only play a role at interphase, but also could be retained in mitotic chromosomes and could be re-used in the interphase chromatin loops of the new cell cycle. The model of Fig 2 explains a most remarkable property of chromosome architecture through the cell cycle, namely, reversibility, *i*.*e*., the fact that at the end of mitosis, the original interphase chromatin loop structure can be precisely recovered thanks to the retention of architectural proteins. Needless to say, the process is possible because it relies on unchanged genome sequences and on the consequent conserved locations of protein binding sites (see Table 3).

**Table 3:** Chromosome architecture changes through the cell cycle.

1. Opening of interphase domains and sub-domains into an extended configuration, probably a 10-nm “beads-on-a-string”, structure.
2. Folding of the extended configuration into 30-nm fiber loops demarcated by the architectural proteins (CTCF and cohesin sub-units) of interphase chromatin that are retained in mitotic chromosomes. Early prophase bands correspond to alternating GC-rich and GC-poor isochores consisting in single- or multiple- 30-nm loops.
3. Coalescence of single-isochore bands into multiple-isochore bands: Prophase to Prometaphase to Metaphase

The changes in chromosome architecture from interphase to mitosis are reversible.

**Fig 7:**
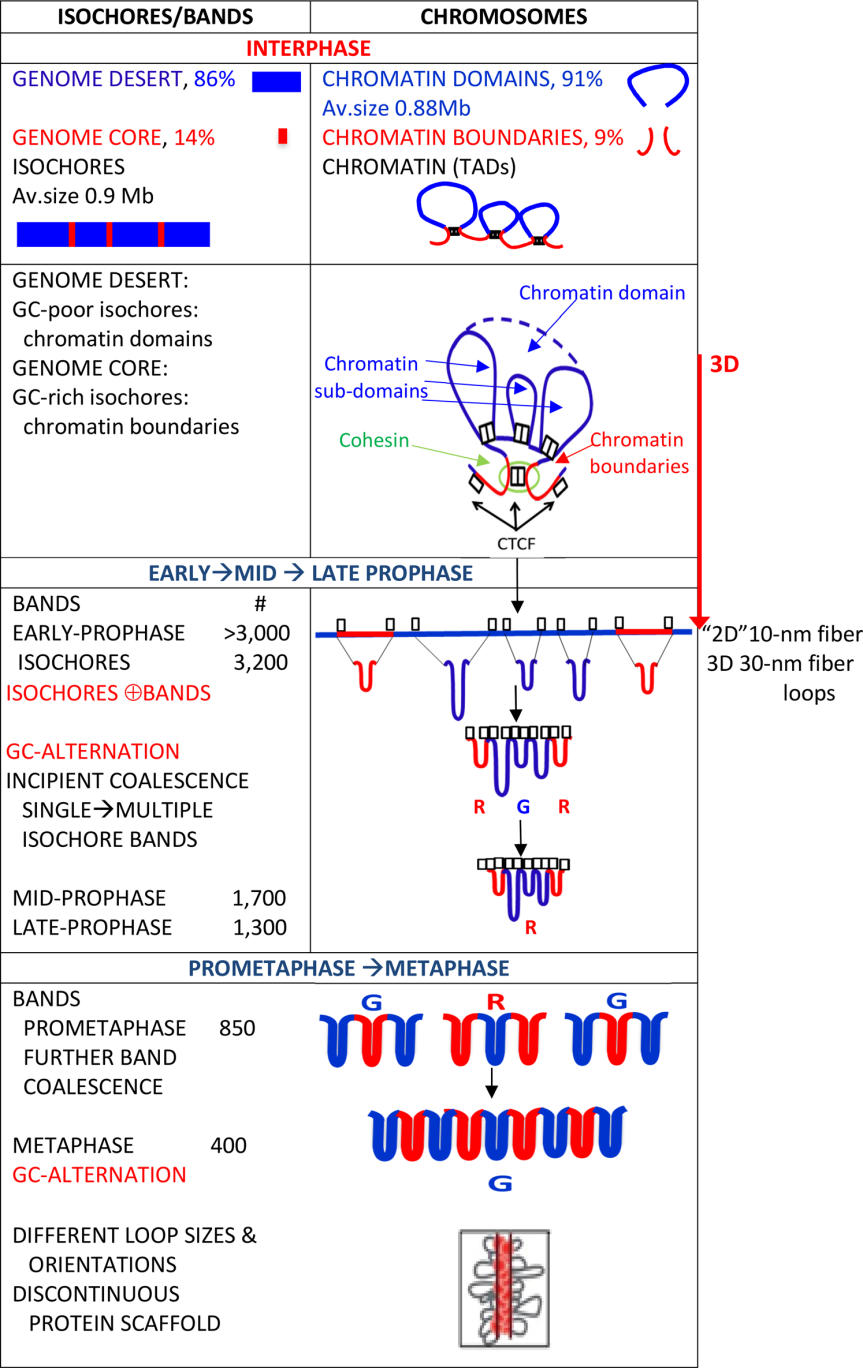
Isochores, chromosomal bands and chromosome architecture. Interphase: See legends of Figs 1C and 2, for the top and bottom panels respectively. Prophase: See legend of Fig 2. The R band of prophase coalesces with two flanking G bands producing a G band. Prometaphase to Metaphase: The multiple-isochore prometaphase bands coalesce further into metaphase bands (see legend of Fig 5C). The central R band of prophase coalesces with two G bands giving rise to a larger G band. The 30-nm loops have different sizes and orientations (the figure is from ref. [64]); the protein scaffold is discontinuous (see Text).

As far as the results summarized the link between chromosome architecture and genome organization is concerned, we already knew that correlations existed between 1) the GC levels of coding and non-coding sequences within the large, compositionally fairly homogeneous DNA segments that were called isochores; and 2) the GC levels of isochores and all tested properties of the genome. These findings supported the idea that the genome is an integrated ensemble. A new, important point is now added to these correlations, that were called the genomic code, by finding that correlations also exist between the compositional properties of isochores and the structural properties of chromosomes through the cell cycle (see Figs 2 and 7). The most remarkable correlation is that between the architecture of interphase chromatin and the isochore organization of the genome (K. Jabbari and G. Bernardi, paper in preparation) because this new point considerably extends the significance of the genomic code and leads to a unifying view of genome organization and chromosome architecture.

In general terms, the present results fulfill the old prophecy that “order must be in chromosomes” [91], support the notion that isochores represent “a fundamental level of genome organization” [92], and represent a conceptual step forward in our understanding of the eukaryotic genome.

## Supporting Information

S1 Table Isochore families in the human genome. (DOCX)
