# Supplementary material for: Chromosome Architecture and Genome Organization
Source: PLoS One. 2015 Nov 30;10(11):e0143739. doi: 10.1371/journal.pone.0143739 (PMC4664426; doi:10.1371/journal.pone.0143739)
Supplement: S1 Table — (DOCX) [file pone.0143739.s001.docx]

| **S1 Table. Isochore families in the human genome ^(a)^** | | | | |
| --- | --- | --- | --- | --- |
|  | Amount  % | Maxima  GC | Minima  GC | Ranges  GC |
| L1 | 19 | 35.5 |  | 33-37 |
|  |  |  | 37 |  |
| L2 | 36 | 38.7 |  | 37-41 |
|  |  |  | 41 |  |
| H1 | 31 | 43.0 |  | 41-46 |
|  | **86** |  | 46 |  |
| H2 | 11 | 48.5 |  | 46-53 |
|  |  |  | 53 |  |
| H3 | 3 | 55.0 |  | 53-59 |
|  | **14** |  |  |  |

(a) Compiled from data of ref. [18]. Values in bold are total amounts of L1+L2+H1 and H2+H3 families, the genome desert and the genome core. The GC ranges of isochore families increase from 4% for L1 and L2, to 5% for H1, to 6-7% for H2 and H3 families. The overall genome GC range is 26%. It should be noted that, at a size of 100Kb, the average standard deviation of GC within isochore families of the human genome reaches a plateau value of ~1% GC for 85% of the genome and ~2% GC for the remaining 15% GC-rich isochore families, while the average standard deviation of the genome is 4.5%-5% GC [16].
